# Exploring mechanisms of sex differences in longevity: lifetime ovary exposure and exceptional longevity in dogs

**DOI:** 10.1111/j.1474-9726.2009.00513.x

**Published:** 2009-12

**Authors:** David J Waters, Seema S Kengeri, Beth Clever, Julie A Booth, Aimee H Maras, Deborah L Schlittler, Michael G Hayek

**Affiliations:** 1 Center for Exceptional Longevity Studies, Gerald P. Murphy Cancer FoundationWest Lafayette, IN, USA; 2 The Department of Veterinary Clinical Sciences and The Center on Aging and the Life Course, Purdue UniversityWest Lafayette, IN, USA; 3 P&G Pet CareLewisburg, OH, USA

**Keywords:** anti-aging, estrogen, ovarian conservation, ovariohysterectomy, sex difference in health

## Abstract

To move closer to understanding the mechanistic underpinnings of sex differences in human longevity, we studied pet dogs to determine whether lifetime duration of ovary exposure was associated with exceptional longevity. This hypothesis was tested by collecting and analyzing lifetime medical histories, age at death, and cause of death for a cohort of canine ‘centenarians’– exceptionally long-lived Rottweiler dogs that lived more than 30% longer than average life expectancy for the breed. Sex and lifetime ovary exposure in the oldest-old Rottweilers (age at death, ≥ 13 years) were compared to a cohort of Rottweilers that had usual longevity (age at death, 8.0–10.8 years). Like women, female dogs were more likely than males to achieve exceptional longevity (OR, 95% CI = 2.0, 1.2–3.3; *P*= 0.006). However, removal of ovaries during the first 4 years of life erased the female survival advantage. In females, a strong positive association between ovaries and longevity persisted in multivariate analysis that considered other factors, such as height, body weight, and mother with exceptional longevity. A beneficial effect of ovaries on longevity in females could not be attributed to resistance against a particular disease or major cause of death. Our results document in dogs a female sex advantage for achieving exceptional longevity and show that lifetime ovary exposure, a factor not previously evaluated in women, is associated with exceptional longevity. This work introduces a conceptual framework for designing additional studies in pet dogs to define the ovary-sensitive biological processes that promote healthy human longevity.

Female survival advantage is well documented in certain mammalian species, most notably humans (Austad, 2006). This translates into a greater likelihood that women will live to 100 years, outnumbering men by approximately 4:1 (Terry *et al.*, 2008). Little progress, however, has been made to elucidate the mechanisms of sex differences in human longevity. Transplantation of mouse ovaries from young donors into ovariectomized female mice increased life expectancy proportional to age of the recipient (Cargill *et al.*, 2003). But the mouse, biogerontology’s most trusted mammalian workhorse, is not likely the most trustworthy mimic of the sex differences in human longevity, because female mice are typically outlived by their male counterparts (Turturro *et al.*, 2002; Austad, 2006).

Whether ovary removal early in life can re-set longevity parameters in humans has not been evaluated, as few young women undergo ovariectomy. In contrast, a large percentage of pet dogs undergo elective ovariectomy at different ages throughout the life course, creating a research opportunity to study the dose–response between endogenous ovarian function and longevity. Here, by studying pet dogs living in the same environment as humans (Waters & Wildasin, 2006), we test the hypothesis that lifetime ovary exposure is significantly associated with exceptional longevity.

A database was established to construct lifetime medical histories for a cohort of 119 oldest-old Rottweiler dogs living in North America. These pet dogs lived with their owners and females underwent elective ovariectomy at different ages throughout the life course. Information on medical history, age at death and cause of death was collected by questionnaire and telephone interviews with pet owners and veterinarians as previously reported (Cooley *et al.*, 2003). Rottweilers with exceptional longevity lived ≥ 13 years, i.e. more than 30% longer than average life expectancy for the breed (9.4 years). In each case, date of birth obtained from American Kennel Club registration records was used to validate exceptional longevity. Sex (female:male) and lifetime duration of ovary exposure in the oldest-old dogs were compared with information collected from another cohort of 186 Rottweilers in the same catchment area that had usual longevity (age at death 8.0–10.8 years).

Like women, female dogs were more likely than males to achieve exceptional longevity (OR, 95% CI = 2.0, 1.2–3.3; *P*= 0.006). However, removal of ovaries during the first 4 years of life (i.e. median age at ovariectomy) erased the female survival advantage over males (OR, 95% CI = 1.2, 0.7–2.2; *P*= 0.55). In females that retained their ovaries for more than 4 years, likelihood of exceptional longevity increased to more than three times that of males (OR, 95% CI = 3.2, 1.8–5.7; *P*< 0.0001).

To further define the dose–response relationship between ovaries and longevity, we focused our analysis on the 83 exceptional longevity females and 100 usual longevity females (Table 1). This enabled us to address the following question: Is the duration of ovary exposure during the first 8 years of life associated with an increased likelihood of achieving exceptional longevity? When females from the exceptional longevity and usual longevity cohorts were combined then subdivided into tertiles based upon ovary exposure during the first 8 years of life, dogs with the longest ovary exposure (6.1–8.0 years) were 3.2 times more likely to reach exceptional longevity than dogs with shortest exposure (*P*= 0.002) (Table 2; Supporting Fig. S1). In multivariate analysis, the association between increasing ovary exposure and exceptional longevity remained strong even after considering other factors that might influence longevity, such as height, body weight and whether mother achieved exceptional longevity (Table 2).

**Table 2:** Endogenous ovary exposure and likelihood of exceptional longevity in female Rottweiler dogs

	Duration of ovary exposure during the first 8 years of life (tertiles)		
All females	1 shortest	2	3 longest
Univariate			
Odds ratio^1^ (95% CI)	1.0	1.6 (0.8–3.4)	3.2 (1.6–6.7)
Range of ovary exposure (years)	0.4–2.0	2.1–6.0	6.1–8.0
Number of dogs	65	57	61
Multivariate^2^			
Odds ratio (95% CI)	1.0	2.4 (0.8–7.2)	4.6 (1.3–16.2)
Range of ovary exposure (years)	0.4–2.0	2.1–6.0	6.1–8.0
Number of dogs	28	28	26
Bone cancer excluded^3^			
Odds ratio (95% CI)	1.0	2.5 (1.1–5.8)	3.9 (1.6–9.3)
Range of ovary exposure (years)	0.4–2.4	2.5–6.5	6.6–8.0
Number of dogs	45	45	44
All cancer excluded^4^			
Odds ratio (95% CI)	1.0	4.0 (1.3–12.2)	9.7 (2.3–40.7)
Range of ovary exposure (years)	0.4–3.1	3.2–7.0	7.1–8.0
Number of dogs	27	30	23

1 Odds ratios were considered significant if 95% confidence interval did not include 1.0.

2 Multivariate odds ratio for 82 females adjusted for height, adult body weight, and mother reaching exceptional longevity. In stepwise regression, in addition to duration of ovary exposure, a second variable, mother reaching exceptional longevity (OR, 95% CI = 8.3, 1.0–67.4), was selected suggesting a strong familial clustering of long-lived individuals.

3 For this analysis, the 44 female dogs whose cause of death was bone cancer were excluded. Appendicular bone sarcoma was the most frequently reported cause of mortality in Rottweilers with usual longevity (8.0–10.8 years).

4 For this analysis, 98 female dogs whose cause of death was cancer of any type were excluded. The odds ratios indicate duration of ovary exposure is significantly associated with exceptional longevity in dogs that succumb to non-cancer causes.

**Table 1:** Characteristics of female Rottweilers in study population

	Usual longevity^1^*N* = 100 dogs	Exceptional longevity^2^*N* = 83 dogs
Age at death^3^ (years), median (IQR)	9.6 (9.0–10.0)	13.6 (13.3–14.3)
Year of birth (range)	1984–2000	1980–1995
Residence		
Geographic distribution	29 states, Canada	27 states, Canada
Number of households^4^	93	79
Lifetime ovary exposure^5^ (years), median (IQR)	2.5 (0.7–6.0)	5.5 (2.0–7.5)
Reproductive history^6^		
Nulliparity in dogs with intact ovaries for ≥ 12 months (%)	26/62 (42)	24/70 (34)
Body weight^7^ (lbs), median (IQR)	90.0 (85.0–100.0)	85.0 (79.2–90.0)
Height^8^ (in), median (IQR)	24.0 (23.0–24.5)	23.5 (22.6–24)
Mother achieved exceptional longevity, *n* (%)^9^		
Yes	1 (3)	11 (22)
No	30 (97)	40 (78)
Cause of death^10^, *n* (%)		
Cancer – all types	73 (73)	25 (32)
Bone sarcoma^11^	38 (38)	6 (8)
Other types	35 (35)	19 (24)
Non-cancer diseases^12^	27 (27)	53 (68)
Gastrointestinal	8 (8)	4 (5)
Musculoskeletal	6 (6)	8 (10)
Cardiovascular	4 (4)	3 (4)
Neurologic	2 (2)	7 (9)
Urologic	1 (1)	2 (3)
Frailty^13^	1 (1)	16 (21)
Other^14^	3 (3)	7 (9)
Unknown	2 (2)	6 (8)

IQR = interquartile range, which indicates the difference between the 1st and 3rd quartiles.

1 Usual longevity cohort represents dogs that died at 8.0–10.8 years, a range surrounding the breed-specific median age at death established in a population-based study of more than 700 Rottweilers (Cooley *et al.*, 2003). The 100 females in the usual longevity group include 34 dogs previously reported by Cooley *et al.* The female:male ratio in the usual longevity cohort was 100:86 (1.26:1). Median (range) age at death for usual longevity males was 9.5 (8.0–10.7 years).

2 Exceptional longevity cohort represents dogs that died at ≥ 13.0 years, which is more than 30% longer than the breed-specific median longevity (9.4 years). The 83 females in the exceptional longevity group include nine exceptionally long-lived dogs reported by Cooley *et al.* (2003). The female:male ratio in the exceptional longevity cohort was 83:36 (2.30:1). Median (range) age at death for exceptional longevity males was 13.6 (13.0–15.5 years).

3 For each dog, age at death was validated using date of birth from American Kennel Club registration records or medical records. The vast majority (> 80%) of dogs underwent elective euthanasia when their quality of life was considered unacceptable by owner.

4 The 183 female dogs in the study population resided in 172 different households. Only 18 owners had more than one dog represented in the study population. Eleven owners had one dog in the usual longevity cohort and one dog in the exceptional longevity cohort.

5 For each dog, duration of ovary exposure is equivalent to age at ovariectomy established in the medical history provided by owners and veterinarians.

6 Thirty-eight usual longevity dogs and 13 exceptional longevity dogs underwent early ovariectomy prior to breeding age, i.e. ovariectomy during first 12 months of life. These dogs were not eligible for reproduction. After excluding these dogs, the table shows a similar percentage of dogs from both groups were not exposed to the ‘reproductive cost’ of offspring.

7 Body weight was obtained from owner questionnaire or medical record, representing when the dog was a healthy 5 to 7-year-old adult.

8 For each dog, height represents shoulder height, the distance measured from ground to shoulder, reported by owner.

9 Data reported here represents only those cases in which information on the longevity of the mother could be validated directly from the owner of the mother. Validated data were available for mothers of 82 of the 183 index females in the study population. A more detailed analysis of the apparent familial clustering of exceptional longevity in these dogs is in progress.

10 For each dog, cause of death was determined by reviewing medical records and medical histories provided by veterinarians and owners. Few causes of death were verified by necropsy and therefore the reliability of these data is likely comparable to that of human mortality studies based on death certificates. There is no reason to suspect that the cause of death was preferentially misclassified in dogs with usual longevity vs. dogs with exceptional longevity, or misclassified on the basis of ovarian hormone exposure as classification of cause of death was made by investigators (DJW, AHM) blinded to age at ovariectomy. In eight of 183 dogs (two usual longevity, six exceptional longevity), cause of death could not be ascertained from the clinical data.

11 Bone cancer (appendicular bone sarcoma) was diagnosed based upon physical examination and radiographs. In some cases, histologic confirmation was made by pathologic examination of tissues obtained at biopsy or necropsy.

12 Death caused by non-cancer diseases was subdivided into five major categories on the basis of frequency: gastrointestinal (e.g. intestinal perforation; inflammatory bowel disease); musculoskeletal (e.g. severe arthritis); cardiovascular (e.g. congestive heart failure); neurologic (e.g. compressive myelopathy due to intervertebral disk herniation; seizures); and urologic (e.g. chronic renal failure).

13 Death was attributed to frailty in those dogs whose owners and veterinarians reported death or euthanasia associated with a combination of age-related disabilities, including deficits in mobility, cognition, hearing, eyesight and inability to maintain body weight.

14 Dogs in this category included those whose cause of death was attributed to less common conditions: hematologic, endocrine, hepatobiliary, or respiratory diseases; environmental causes (e.g. heat stroke); and dogs that died in their sleep without recognized illness.

Finally, we evaluated whether the survival advantage in females with intact ovaries could be explained by a protective effect of ovaries against a particular disease. In Rottweilers with usual longevity, the major cause of death and major death category were bone sarcoma and cancer-all types, accounting for 38% and 73% deaths, respectively. We found that, after excluding bone cancer deaths or all cancer deaths, the strong association between intact ovaries and exceptional longevity persisted. After excluding all cancer deaths, females who kept their ovaries during the first 7 years of life (i.e. highest tertile of ovary exposure) were more than nine times more likely to reach exceptional longevity than females with shortest ovary exposure (*P*= 0.001) (Table 2).

Our results show that in Rottweiler dogs, like in humans, there is a strong female sex advantage for reaching exceptional longevity. Importantly, the longevity advantage over males is abolished in females that undergo early or mid-life ovarian removal. To our knowledge, this is the first demonstration that lifetime ovary exposure is significantly associated with exceptional longevity in any mammalian species.

By studying dogs that underwent elective ovariectomy at different ages, we were able to probe the dose–response relationship between endogenous ovarian function and exceptional longevity. A possible longevity-promoting effect of ovaries in exceptionally long-lived dogs in this study is supported by data from another cohort of pet dogs that we have investigated (Supporting Fig. S2). In a population of 237 female Rottweiler dogs who died at 1.3–12.9 years, females that had intact ovaries for the first 4.5 years of life had 37% lower mortality than females that underwent elective ovariectomy before 4.5 years, i.e., median age at ovariectomy (hazard ratio, 95% CI = 0.63, 0.49–0.82; *P* < 0.0001 log-rank test). The research strategy advanced here by our group – using a national population of registered, pure-bred dogs of a single breed, carefully quantitating the duration of ovary exposure rather than binning dogs into a category of either ‘intact’ or ‘ovariectomized’– lessens the likelihood of misclassification bias that likely plagued previous dog studies (Bronson, 1982; Michell, 1999).

Recognizing that observed associations between exposures and outcomes may not necessarily be causal, we explored alternative, non-causal explanations for the association between ovaries and exceptional longevity. But, we found no evidence that factors which may influence a pet owner’s decision on age at ovariectomy – for example, earlier ovariectomy in dogs with substandard conformation or delayed ovariectomy to obtain more offspring in daughters of long-lived mothers – can adequately account for the strong association (Supporting Appendix S1). Further, our results mirror the recent findings from more than 29 000 women in the Nurses’ Health Study (Parker *et al.*, 2009). In that study, women who had elective hysterectomy with ovary sparing had lower overall mortality than those who underwent hysterectomy with ovariectomy. Notably, the benefit of keeping ovaries experienced by women under 50 years was attributable to decreased cardiovascular and cancer mortality (Parker *et al.*, 2009). Taken together, the findings from dogs and women support the hypothesis that early life physiological influences, such as ovarian hormones, lay the foundation for adult health outcomes including longevity. Further testing of the ovary-longevity hypothesis should utilize experimental designs that capture the broad range of lifetime ovary exposure seen in the pet dog population so that the critical windows of ovary exposure can be better defined.

Specific mechanisms have been proposed by which ovaries might promote longevity, including estrogen-induced enhanced immune response (Austad, 2006; Straub, 2007) and protection against oxidative stress (Borras *et al.*, 2003). We could not attribute the ovary-associated longevity advantage in dogs to resistance against a particular disease. Instead, we observed a robust ovarian association with longevity that was *independent of cause of death*, suggesting that a network of processes regulating the intrinsic rate of aging is under ovarian control. Future studies in dogs and women are warranted to define specific ovarian longevity factors and to identify ovary-sensitive biological processes that promote healthy longevity. Pet dogs should provide a tractable mammalian model to investigate the mechanisms of how ovaries orchestrate extended longevity in both species.

## Supporting Information

**Fig. S1** Life course analysis of ovary exposure during the first 8 years of life in dogs with usual longevity versus exceptional longevity.

**Fig. S2** Increased lifetime ovary exposure is associated with increased longevity in 237 female Rottweiler dogs.

**Appendix S1** Additional analysis used to explore alternative, non-causal explanations for the association between lifetime ovary exposure and exceptional longevity.

As a service to our authors and readers, this journal provides supporting information supplied by the authors. Such materials are peer-reviewed and may be re-organized for online delivery, but are not copy-edited or typeset. Technical support issues arising from supporting information (other than missing files) should be addressed to the authors.
