# Supplementary material for: Exploring mechanisms of sex differences in longevity: lifetime ovary exposure and exceptional longevity in dogs
Source: Aging Cell. 2009 Dec;8(6):752–5. doi: 10.1111/j.1474-9726.2009.00513.x (PMC2805875; doi:10.1111/j.1474-9726.2009.00513.x)
Supplement: Supplementary file 1 [file ace0008-0752-SD1.ppt]

## Slide 1
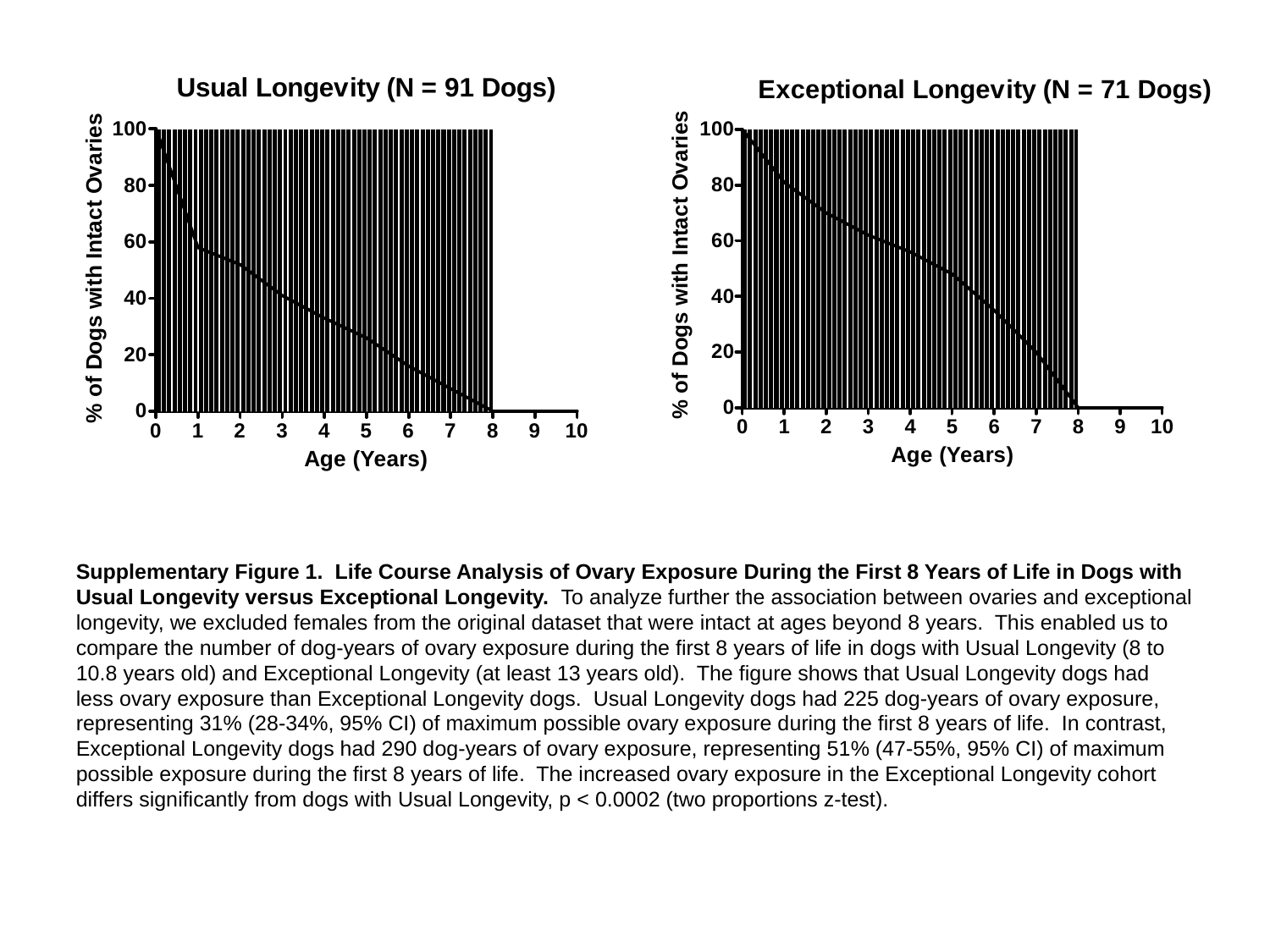

Supplementary Figure 1. Life Course Analysis of Ovary Exposure During the First 8 Years of Life in Dogs with Usual Longevity versus Exceptional Longevity. To analyze further the association between ovaries and exceptional longevity, we excluded females from the original dataset that were intact at ages beyond 8 years. This enabled us to compare the number of dog-years of ovary exposure during the first 8 years of life in dogs with Usual Longevity (8 to 10.8 years old) and Exceptional Longevity (at least 13 years old). The figure shows that Usual Longevity dogs had less ovary exposure than Exceptional Longevity dogs. Usual Longevity dogs had 225 dog-years of ovary exposure, representing 31% (28-34%, 95% CI) of maximum possible ovary exposure during the first 8 years of life. In contrast, Exceptional Longevity dogs had 290 dog-years of ovary exposure, representing 51% (47-55%, 95% CI) of maximum possible exposure during the first 8 years of life. The increased ovary exposure in the Exceptional Longevity cohort differs significantly from dogs with Usual Longevity, p < 0.0002 (two proportions z-test).

## Slide 2
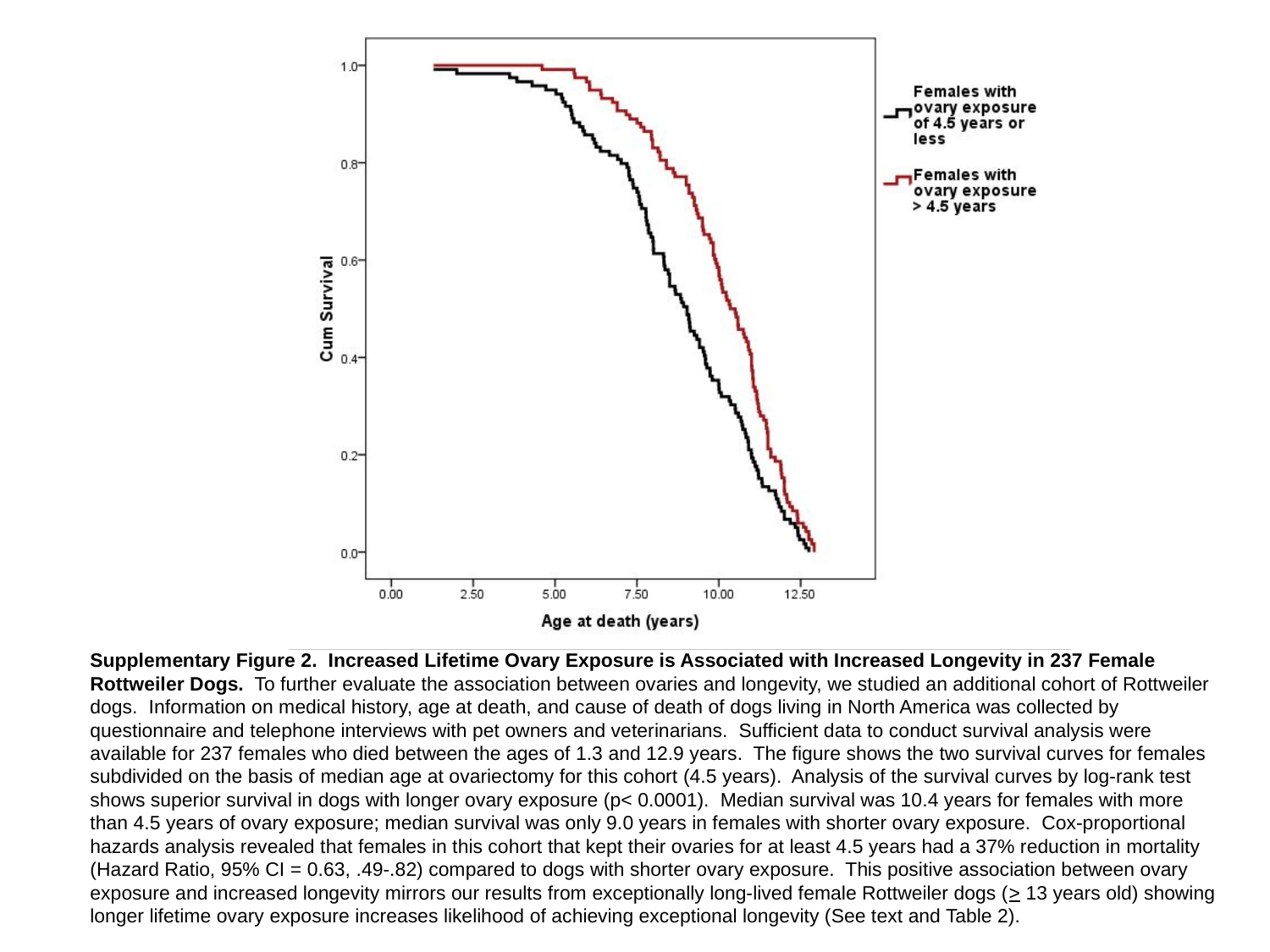

Supplementary Figure 2. Increased Lifetime Ovary Exposure is Associated with Increased Longevity in 237 Female Rottweiler Dogs. To further evaluate the association between ovaries and longevity, we studied an additional cohort of Rottweiler dogs. Information on medical history, age at death, and cause of death of dogs living in North America was collected by questionnaire and telephone interviews with pet owners and veterinarians. Sufficient data to conduct survival analysis were available for 237 females who died between the ages of 1.3 and 12.9 years. The figure shows the two survival curves for females subdivided on the basis of median age at ovariectomy for this cohort (4.5 years). Analysis of the survival curves by log-rank test shows superior survival in dogs with longer ovary exposure (p< 0.0001). Median survival was 10.4 years for females with more than 4.5 years of ovary exposure; median survival was only 9.0 years in females with shorter ovary exposure. Cox-proportional hazards analysis revealed that females in this cohort that kept their ovaries for at least 4.5 years had a 37% reduction in mortality (Hazard Ratio, 95% CI = 0.63, .49-.82) compared to dogs with shorter ovary exposure. This positive association between ovary exposure and increased longevity mirrors our results from exceptionally long-lived female Rottweiler dogs (> 13 years old) showing longer lifetime ovary exposure increases likelihood of achieving exceptional longevity (See text and Table 2).
