# Supplementary material for: Exploring mechanisms of sex differences in longevity: lifetime ovary exposure and exceptional longevity in dogs
Source: Aging Cell. 2009 Dec;8(6):752–5. doi: 10.1111/j.1474-9726.2009.00513.x (PMC2805875; doi:10.1111/j.1474-9726.2009.00513.x)
Supplement: Supplementary file 2 [file ace0008-0752-SD2.doc]

**Appendix 1. Additional Analysis to Explore Alternative, Non-Causal Explanations for the Association Between Lifetime Ovary Exposure and Exceptional Longevity**

Since the exposure in this investigation (duration of intact ovaries) was not randomized in this observational study, we evaluated whether factors which might influence a pet owner’s decision on age at ovariectomy could account for the strong positive association between ovaries and longevity.

First, we evaluated the following scenario: If owners recognize a strong familial component to exceptional longevity, then daughters of vigorous females may be kept intact longer for the sake of breeding. This would be observed as a positive association between duration of ovary exposure and likelihood of exceptional longevity. To evaluate this “Special Mom Hypothesis”, we examined whether there were differences in 4 parameters (median age at spay; percent of dogs spayed less than 12 months (reproductively ineligible); percent nulliparity in dogs that kept their ovaries greater than 12 months; number of litters) between 3 subgroups of females: (1) Exceptional Longevity (**EL**) females with exceptional longevity mother (N=11); (2) Exceptional Longevity females with usual longevity mother (N=40); and (3) Usual Longevity (**UL**) females with usual longevity mother (N=30).

If the special mom effect were strongly operational, we would expect the Exceptional Longevity females with exceptional longevity mother to have intact ovaries longer and have more litters than Exceptional Longevity females with usual longevity mother. However, we found no significant differences in the percent spayed less than 12 months (7.5% versus 9%), percent nulliparity if spayed after 12 months (41% vs. 27%), number of litters (2.35 vs. 2.33),

or median age at ovariectomy (5.0 years vs. 6.0 years) (**see** **Table below**).

|  | **1st subgroup**  **EL females** with EL mother  n=11 | **2^nd^ subgroup**  **EL** females with UL mother  n=40 | **3^rd^ subgroup**  **UL** females with UL mother  n=30 | **p-values**  **1^st^ vs. 2^nd^ subgroup** |
| --- | --- | --- | --- | --- |
| **Median age at ovariectomy** | 6.0 yrs | 5.0 yrs | 1.9 yrs | p=0.92 |
| **% of ovariectomy < 12 months old** | 9% | 7.5% | 40% | p=1.00 |
| **% Nulliparity if ovariectomy >12 months old** | 27% | 41% | 29.6% | p=0.41 |
| **Number of litters (mean)** | 2.33 | 2.35 | 1.71 | p=0.98 |

In contrast, comparing the Usual Longevity and Exceptional Longevity females that each had usual longevity mothers revealed significant differences in 2 parameters that support our ovaries-longevity hypothesis: percent of dogs that underwent ovariectomy less than 12 months (40% in Usual Longevity vs. only 7.5% in the Exceptional Longevity females; **p = .002)** and median age at ovariectomy (1.9 years for Usual Longevity dogs vs. 5.0 years for Exceptional Longevity dogs; **p = .005).**

Next, we further explored the possibility that the owner’s decision to elect ovariectomy could create an artificial association between ovaries and longevity by evaluating the following scenario: Pet owners may select early ovariectomy (less than 4 years) if they recognize certain non-desirable physical traits such as poor hip conformation, joint laxity, eye problems. If these characteristics disadvantage a dog in terms of longevity, then early ovariectomy for physical inadequacies might create an artifactual positive association between keeping ovaries and achieving exceptional longevity. To evaluate this “Poor Physical Conformation Hypothesis”, we gathered information from pet owners on the reasons that prompted ovariectomy. Responses fell into 4 categories: (1) Sterilization/stop estrous cycling; (2) Treatment of breast or uterine disease; (3) Remediation of reproductive problems/cease breeding; and (4) Substandard physical conformation. Substandard physical conformation was reported as the reason that prompted ovariectomy in only 9 females in our study population – 6 Usual Longevity females, 3 Exceptional Longevity females. After excluding these 9 dogs from the 183 females, we found the Odds Ratios (95% CI) for likelihood of achieving exceptional longevity were 1.0, 1.7 (0.8-3.6), and 3.3 (1.5-7.0) in the low, medium, and high tertiles of ovary exposure **(see Table below**).

|  | **Duration of Ovary Exposure**  **During the First 8 Years of Life (tertiles)** | | |
| --- | --- | --- | --- |
| **Excluding Substandard**  **Conformation Females** | **1**  **shortest** | **2** | **3**  **longest** |
| **Univariate** |  |  |  |
| Odds Ratio**^*^**  (95% CI) | 1.0 | 1.7  (0.8-3.6) | 3.3  (1.5-7.0) |
| Range of ovary exposure | 0.4-2.0 yrs | 2.1-6.2 yrs | 6.3-8.0 yrs |
| Number of dogs | 59 | 56 | 59 |

**^*^**Odds Ratios were considered significant if 95% confidence interval (CI) did not include 1.0.

These results are essentially unchanged from the results of 183 female dogs shown in **Table 2 of the manuscript**, where the Odds Ratios are 1.0, 1.6, and 3.2 for tertiles of ovary exposure.
